# Ectopic pregnancy-related severe maternal morbidity in Shenzhen: a sentinel indicator for gaps in early pregnancy care systems

**DOI:** 10.7189/jogh.16.04331

**Published:** 2026-10-09

**Authors:** Yu Dai, Qiaoxuan Wu, Litong Zhu, Weichun Yang, Ping Jin, Shuyan Jin, Fuying Tian, Xiaotian Li

**Affiliations:** 1 Department of Obstetrics and Gynecology, Shenzhen Maternity and Child Healthcare Hospital, Women and Children's Medical Center, Southern Medical University, Shenzhen, China; 2 Shenzhen School of Clinical Medicine, Southern Medical University, Shenzhen, China; 3 Shenzhen Clinical Research Center for Obstetrics & Gynecology and Reproductive System Diseases, Shenzhen, China.; 4 Shenzhen Key Laboratory of Maternal and Child Health and Diseases, Shenzhen, China; 5 The Obstetrics &Gynecology Hospital Fudan University, Shanghai, China

**Keywords:** ectopic pregnancy, severe maternal morbidity, sentinel indicator, healthcare system gaps

## Abstract

**Background:**

Despite China’s progress in reducing maternal mortality, severe maternal morbidity in early pregnancy, particularly ectopic pregnancy, remains a critical challenge in urban settings like Shenzhen. This study examines the clinical presentation scenarios, types, and sociodemographic compositions of ectopic pregnancy -related severe maternal morbidity to map potential structural vulnerabilities within the maternal healthcare continuum and propose interventions aligned with China’s ‘Healthy Motherhood 2030’ agenda.

**Methods:**

A retrospective analysis used 2023 data from Shenzhen’s Maternal and Child Health Care Management Information System, covering 66 maternity hospitals. A total of 245 ectopic pregnancy-related severe maternal morbidity cases were identified using standard WHO near-miss criteria. Statistical analyses evaluated the descriptive distributions and baseline profiles of clinical outcomes across different initial resuscitation settings.

**Results:**

Among 154,000 live births, 1,075 severe maternal morbidity cases (0.70%) included 257 (23.9%) ectopic pregnancy-related emergencies. After exclusions, we analysed 245 cases: 67.35% multiparous women, 31.84% had a junior high education or lower and 95% without prenatal care. Ectopic pregnancy-severe maternal morbidity occurred mostly at home (46.12%), followed by outpatient/emergency (30.20%) and inpatient (23.67%) settings. Among home/emergency presentations, 83.3% of women had lower education and 80.4% were employed. Tubal isthmus pregnancies (42.45%) dominated home-based emergencies, while atypical sites were frequent in clinical settings. Advanced severe complications occurred in only seven cases, limiting meaningful statistical comparisons across settings.

**Conclusions:**

In a high-resource, low-MMR urban environment, ectopic pregnancy-related emergencies account for nearly one-quarter of the total SMM burden, with acute crises often concentrated in pre-hospital or frontline care settings. These descriptive patterns suggest that maternal safety strategies should consider shifting focus toward enhancing community health literacy and rapid-response integration at the primary care level. Forward-looking interventions – such as mobile health platforms, clinical simulation training, and formalised early pregnancy emergency networks – warrant prospective evaluation as potential operational pathways to bridge the identified gaps.

Global advancements have significantly reduced the maternal mortality ratio (MMR) [1], primarily due to improved prenatal care and better management of late-pregnancy complications. In China, a well-established three-tier maternal and child health network has played a crucial role in achieving a remarkably low MMR [2], especially in cities such as Shenzhen, where it has fallen below five per 100,000 live births [3]. This achievement, while commendable, emphasises a new challenge: ensuring the safety of women who survive severe, life-threatening complications. Severe maternal morbidity [4–6] (SMM), defined as a critical near-miss event requiring urgent intervention, has emerged as a vital measure of healthcare system effectiveness and a warning signal for potential fatalities. Thus, understanding SMM is essential for strengthening maternal health in regions where mortality rates are already low.

In this context, the epidemiological landscape of SMM has evolved. Historically, postpartum haemorrhage [7] was one of the primary causes. However, in places like Shenzhen, early pregnancy emergencies, particularly ectopic pregnancy (EP), have become prevalent [8]. This shift reveals a significant gap in maternal health services, as routine antenatal care typically commences after 12 weeks of gestation, leaving early pregnancy complications unmonitored. Ectopic pregnancy [9], characterised by embryo implantation outside the uterus, carries a high risk of rupture [10,11] and sudden hemodynamic collapse [12], making it an urgent healthcare concern [13].

The outcomes associated with SMM related to EP are not determined by the treatability of the condition but rather by delays in the survival process [14]. NHS data indicates 30 cases of EP were misdiagnosed in 2017–2018, leading to substantial patient harm [15]. Therefore, investigating the initial resuscitation of patients experiencing haemorrhagic shock due to a ruptured EP offers valuable insights for systemic evaluation. Whether this first response occurs at home, in a primary care clinic, in an emergency department, or at a tertiary centre, it reveals vulnerabilities within the maternal safety infrastructure. For instance, shock occurring at home indicates insufficient public health education and poor symptom recognition [16,17]. Delays or misdiagnoses [18,19] at the first point of healthcare contact suggest inadequacies in diagnostic skills [20], accessible ultrasound, or referral protocols [21]. Even within specialised centres, suboptimal outcomes may highlight inefficiencies in emergency responses or a lack of expertise in managing complex cases [22]. Consequently, EP-related shock serves as a strategic indicator, highlighting potential systemic weaknesses from community-level care to tertiary healthcare settings.

This study, conducted in the megacity of Shenzhen – renowned for its advanced medical resources and a substantial migrant population [23] of women of reproductive age – aims to leverage this perspective. By analysing cases of haemorrhagic shock due to ectopic pregnancy, we intend to explore the clinical characteristics, timing, and outcomes related to initial resuscitation scenarios. The objective is to use EP-related shock as a tracer condition to map and explore potential vulnerabilities within the maternal healthcare continuum. Our findings aim to inform evidence-based strategies that enhance the rapid-response chain, ultimately further reducing SMM and supporting China’s ‘Healthy Mother 2030’ [24] initiative.

## METHODS

### Data source and participants

This retrospective study used data from the Shenzhen Maternal and Child Health Care Management Information System, a government-mandated registry that captures obstetric outcomes across all 66 maternity hospitals in Shenzhen, China. Data were extracted for the period from 1 January to 31 December 2023, using ICD-10 codes (Data Sheet 7 in the **Online Supplementary Document**) and covering all ten districts: Luohu, Futian, Nanshan, Yantian, Bao’an, Longhua, Longgang, Guangming, Pingshan, and Dapeng New District. The data set included demographic characteristics, gestational weeks at which rescue interventions were initiated, clinical diagnoses, treatments, and outcomes. Severe maternal morbidity (SMM) was defined according to World Health Organization (WHO) standards, which comprise five clinical indicators (such as organ dysfunction, haemorrhagic shock, and eclampsia/pre-eclampsia) and three intervention-based criteria. Cases were extracted from the registry using a strict cross-filtering logic: a patient was identified as an EP-SMM case only if their record simultaneously contained an Ectopic pregnancy code AND an SMM clinical indicator code. The specific SMM indicators and EP indicators adopted in this study are provided in detail (Data Sheet 7 in the **Online Supplementary Document**).

The study protocol was approved by the Institutional Ethics Committee in accordance with the ethical standards of the Declaration of Helsinki (2013 revision) and Good Clinical Practice guidelines. Our study conformed to the Strengthening the Reporting of Observational Studies in Epidemiology (STROBE) reporting guideline for retrospective studies.

### Conditions and measurements

This study focused on patients diagnosed with ectopic pregnancy (EP) complicated by shock, defined as the implantation of an embryo outside the uterine cavity, potentially leading to severe complications such as haemorrhagic shock. We employed ICD-10 codes for ‘ectopic pregnancy’ and ‘SMM’ (Data Sheet 7 in the **Online Supplementary Document**) to determine the ratio of EP-specified SMM and to clarify the incidence and outcomes associated with this condition. Ectopic pregnancies were classified into several types based on clinical and imaging criteria: tubal ectopic pregnancy (the most common), ovarian ectopic pregnancy, abdominal ectopic pregnancy, and cervical ectopic pregnancy.

We collected comprehensive information related to the patients, including demographic details such as age, gravidity, parity, occupation, and educational level. Additional data regarding the ectopic pregnancy included the type, gestational weeks at diagnosis, severity of the condition, and the setting where rescue efforts were initiated. Rescue scenarios represent the specific environment where the life-threatening crisis was first recognised, thereby initiating the emergency resuscitation chain:

1. ‘at home’: specifies that the severe clinical deterioration (*e.g.* sudden agonising abdominal pain, syncope, or collapse) occurred and was first recognised outside of any medical facility. This prompted immediate emergency dispatch or urgent self-transfer to a hospital.

2. ‘outpatient/emergency department’: specifies instances where the patient was already physically present within an outpatient clinic or emergency department when sudden shock developed or was first diagnosed by a frontline clinician, triggering immediate on-site resuscitation.

3. ‘inpatient department’: specifies cases where the patient was already admitted to a hospital ward when tubal rupture and sudden shock occurred under direct inpatient monitoring.

### Statistical analysis

We cleaned and analysed case data using SPSS software, version 22 (Armonk, New York, USA). Count data are expressed as (n, %). The χ^2^ test or Fisher exact probability method was used to compare differences in categorical variables among multiple independent samples. Measurement data are presented as mean (standard deviation). The independent samples *t* test was used to compare differences in continuous variables between two independent groups, while one-way ANOVA was used to compare differences in continuous variables among multiple independent samples. A two-sided *P* < 0.05 was considered statistically significant. Before using the independent samples *t* test and one-way ANOVA, Levene’s test was conducted to assess the homogeneity of variance. For data that do not meet the assumption of homogeneity of variance, the rank-sum test (Mann-Whitney U test) or Welch’s ANOVA will be used. For secondary outcomes or clinical subgroups with extremely low counts, formal hypothesis testing was intentionally bypassed, and analysis was restricted to baseline descriptive reporting.

## RESULTS

### Patient characteristics

In 2023, the total number of live births across all 66 delivery points in Shenzhen was 154,000, with 1,075 cases (0.70%) of severe maternal morbidity (SMM) recorded among the mothers. Of these SMM cases, 257 (23.9%) were classified as ectopic pregnancy (EP)-related emergencies necessitating critical intervention. After excluding 12 cases (nine with undocumented implantation sites and three with incomplete resuscitation records), 245 EP cases (95.2%) met the inclusion criteria for analysis (**Figure 1**).

**Figure 1:**
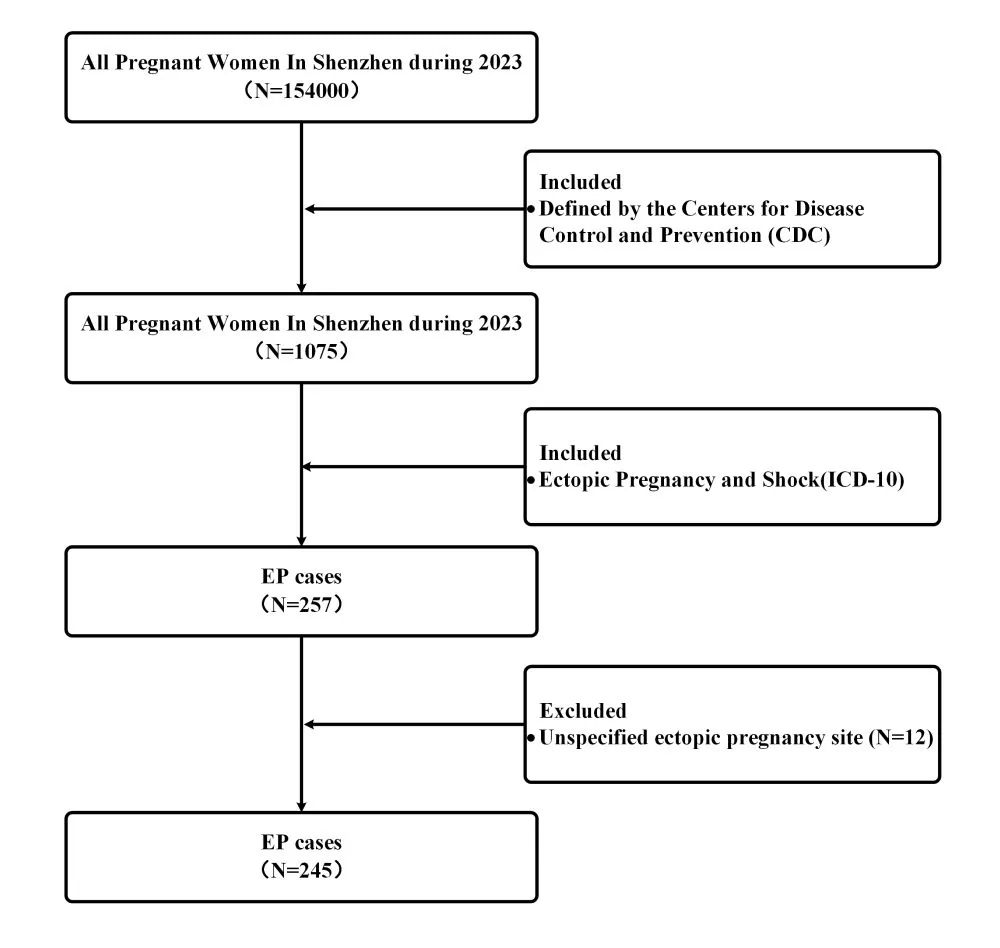
Flowchart of study participant selection.

The demographic characteristics of ectopic pregnancy-related severe maternal morbidity (SMM-EP) patients are provided in detail (**Table 1**). Notably, among the 245 cases of SMM-EP, multiparous women accounted for 67.35%, with a mean age of 31.83 ± 6.03 years. Additionally, 31.84% (n/N = 78/245) of the women had a junior high school education or lower. Over 95% (n/N = 233/245) had not received prenatal care during this pregnancy. The mean gestational age was 6.63 ± 1.90 weeks, with only 16 cases (6.5%) occurring after 10 weeks of gestation. Of the cases, 46.12% (n/N = 113/245) occurred at home, 30.20% (n/N = 74/245) occurred in outpatient/emergency departments, and only 23.67% (n/N = 58/245) took place in inpatient units (**Figure 2**, Panel A).

**Table 1:** Characteristics of EP-SMM*

Baseline data	N = 245
Age, in years, mean ± SD	31.83 ± 6.03
Gestational age, in weeks, mean ± SD	6.63 ± 1.90
Gravidity	
*1*	51 (20.82)
*2 ~ 4*	147 (60.00)
*≥5*	47 (19.18)
Parity	
*0*	80 (32.65)
*1 ~ 3*	158 (64.49)
*≥4*	7 (2.86)
Educational	
*Junior high school or below*	78 (31.84)
*High school*	48 (19.59)
*College*	67 (27.35)
*University*	49 (20.00)
*Masters or above*	3 (1.22)
Occupation	
*Housewife or unspecified*	97 (39.59)
*Worker or farmer*	54 (22.04)
*Merchant*	14 (5.71)
*Staff or cadre*	80 (32.65)
Prenatal healthcare	
*0*	233 (95.10)
*≥1*	12 (4.90)
Different settings	
*Home*	113 (46.12)
*Outpatient or emergency*	74 (30.20)
*Inpatient*	58 (23.67)

EP-SMM – ectopic pregnancy-severe maternal morbidity, SD – standard deviation

*The values are presented as n (%) unless specified otherwise.

**Figure 2:**
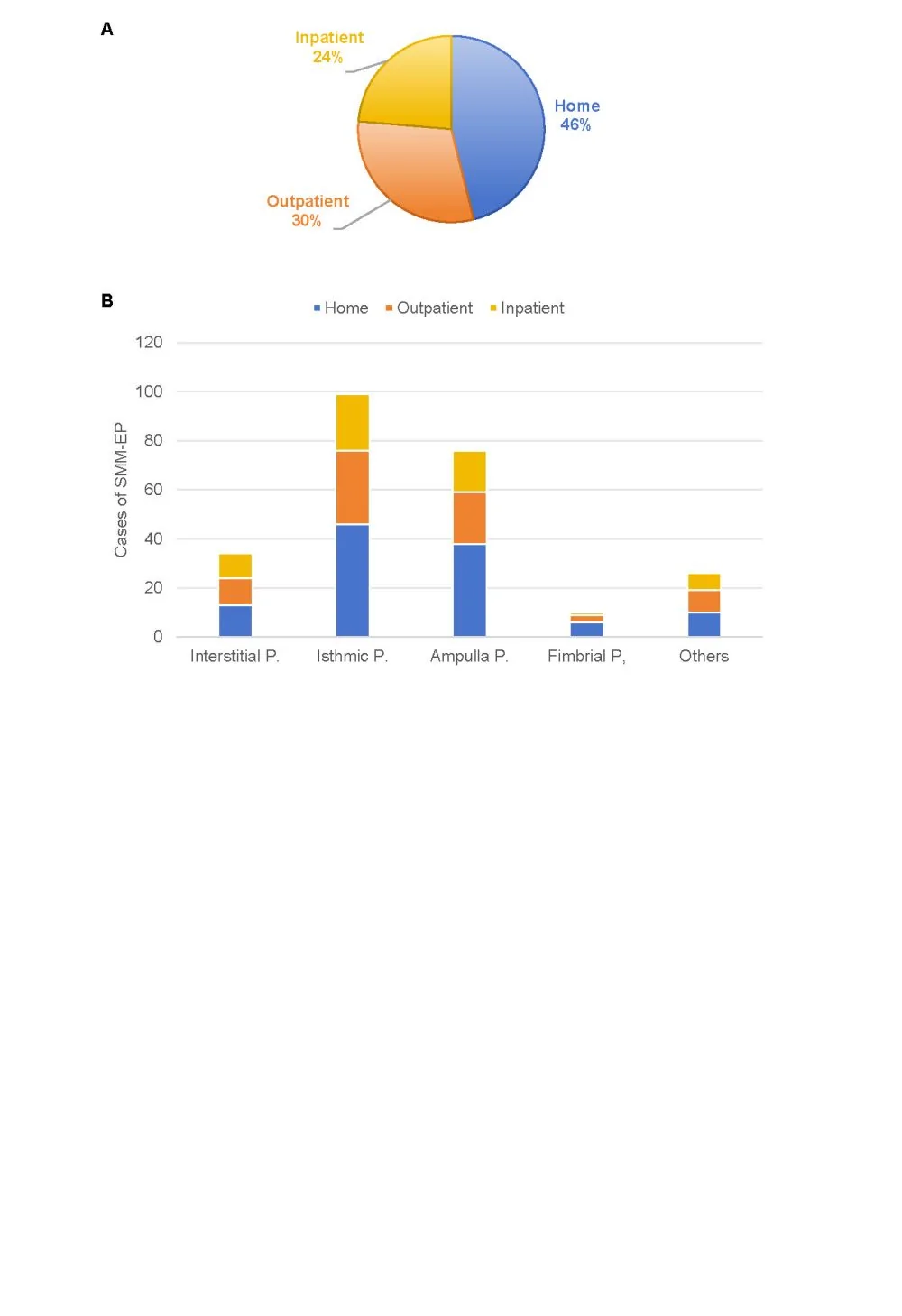
Types of ectopic pregnancy. Panel A. Distribution of ectopic pregnancy cases by setting. Panel B. Anatomical type of ectopic pregnancy by setting.

Indicators of poor prognosis included at least one of the following: DIC, heart failure, pulmonary embolism, or respiratory failure. A total of seven cases were recorded, comprising four with DIC, one with heart failure, one with respiratory failure, and two with pulmonary embolism, one of which presented with DIC alongside respiratory failure.

### Characteristics in different settings

The characteristics of SMM-EP in various settings are summarised in detail (**Table 2**). In terms of sociodemographic composition, women presenting in the home or emergency department settings were characterised by a high proportion of lower educational attainment (junior high school or below, accounting for 83.3%) and a high proportion of employment status (80.4%). Other demographic and clinical factors including maternal age, gravidity, gestational age, frequency of prenatal visits, and anatomical type of ectopic pregnancy, displayed relatively homogeneous baseline distributions across the home, outpatient/emergency department, and inpatient groups.

**Table 2:** Characteristics of SMM-EP In different settings*

Characteristics		Home (n = 113)	Outpatient or emergency (n = 74)	Inpatient (n = 58)	*F/X^2^*	*P-*value
Age, mean ± SD		31.94 ± 5.99	31.89 ± 6.22	31.53 ± 5.98	0.091†	0.913
Gestational age, in weeks, mean ± SD		6.45 ± 1.87	6.80 ± 2.09	6.78 ± 1.71	0.955†	0.386
Gestational age, weeks					6.610‡	0.298
*≤6*	67 (59.29)		32 (43.24)	30 (51.72)		
*7–9*	40 (35.40)		35 (47.30)	25 (43.10)		
*≥10*	6 (5.31)		7 (9.46)	3 (5.17)		
Gravidity					0.453‡	0.979
*1*	22 (19.47)		17 (22.97)	12 (20.69)		
*2 ~ 4*	68 (60.18)		44 (59.46)	35 (60.34)		
*≥5*	23 (20.35)		13 (17.57)	11 (18.97)		
Parity					2.549‡	0.641
*0*	35 (30.97)		25 (33.78)	20 (34.48)		
*1 ~ 3*	73 (64.60)		47 (63.51)	38 (65.52)		
*≥4*	5 (4.42)		2 (2.70)	0 (0.00)		
Educational level						
*Junior high school /below*	35 (30.97)		30 (40.54)	13 (22.41)		
*High school*	23 (20.35)		7 (9.46)	18 (31.03)		
*College*	25 (22.12)		24 (32.43)	18 (31.03)		
*Bachelor*	28 (24.78)		12 (16.22)	9 (15.52)		
*Master and above*	2 (1.77)		1 (1.35)	0 (0.00)		
Occupation						
*Housewife/unspecified*	40 (35.40)		28 (37.84)	29 (50.00)		
*Worker/farmer*	19 (16.81)		24 (32.43)	11 (18.97)		
*Merchant*	7 (6.19)		4 (5.41)	3 (5.17)		
*Staff/cadre*	47 (41.59)		18 (24.32)	15 (25.86)		

SMM-EP – severe maternal morbidity-ectopic pregnancy, SD – standard deviation

*The values are presented as n (%) unless specified otherwise.

†One-way ANOVA

‡χ^2^ test.

### Emergency settings and ectopic pregnancy types

There were no differences in the various characteristics of SMM-EP among the locations of the EP, except concerning prenatal healthcare (**Table 3**; Data Sheet 1 in the **Online Supplementary Document**). Among the cases, the majority of rescue cases of EP occurred in the isthmus of the fallopian tube (99, 40.41%), followed by the ampulla of the fallopian tube (76, 31.02%), with 34 cases in interstitial pregnancies, 10 in fimbrial pregnancies, and 26 cases in other locations. There was no significant correlation between the types of ectopic pregnancy and the occurrence settings (**Figure 2**, Panel B).

**Table 3:** Emergency settings and ectopic pregnancy types

		Home, n = 113, n (%)	Outpatient or emergency, n = 74, n (%)	Inpatient, n = 58, n (%)	*X^2^*	*P-*value
Prenatal healthcare					2.506*	0.779
*0*	108 (95.58)		69 (93.24)	56 (96.55)		
*≥1*	5 (4.42)		5 (6.76)	2 (3.45)		
Types					3.323*	0.921
*Interstitial p.*	13 (11.50)		11 (14.86)	10 (17.24)		
*Ampulla p.*	38 (33.63)		21 (28.38)	17 (29.31)		
*Isthmic p.*	49 (43.36)		32 (40.24)	23 (39.66)		
*Fimbrial p.*	6 (5.31)		3 (4.05)	1 (1.72)		
*Others*	7 (6.19)		7 (9.46)	7 (12.07)		

*χ^2^ test.

### Emergency settings and gestational age

The distribution of gestational age for emergency interventions among different types of ectopic pregnancies is provided in detail (Data Sheet 4 in the **Online Supplementary Document**). For the majority of ectopic pregnancy locations, emergency presentations were concentrated at 6–7 weeks of gestation (**Figure 3**, Panel A). Consistent with this distribution, there was no statistically significant association between the occurrence settings and gestational age. Proportions of all gestational ages occurring at home, outpatient, or emergency department settings were relatively high (**Figure 3**, Panel B).

**Figure 3:**
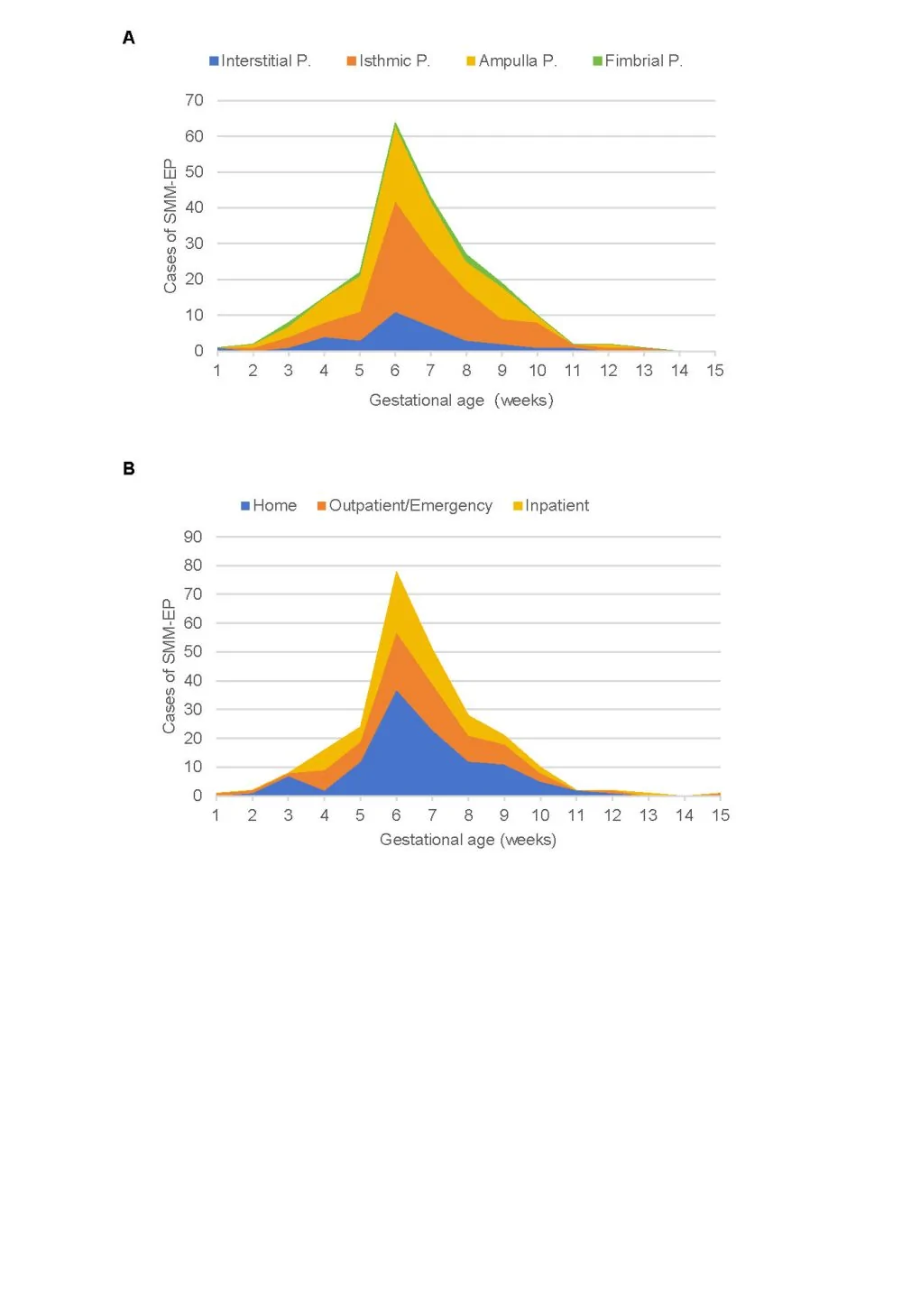
Cases of severe maternal morbidity-ectopic pregnancy in different gestational age. Panel A. Gestational age distribution by anatomical type of ectopic pregnancy locations. Panel B. Gestational age distribution by setting.

### Emergency settings and severe complications

Among the seven patients with severe complications from SMM-EP, three cases occurred at home; one was DIC, one had respiratory failure, and another presented with cardiac and respiratory failure. Two cases that occurred in the emergency department included one with pulmonary embolism and one with DIC. Two inpatient cases were both DIC. Due to the extremely low event count (n = 7), the study lacks sufficient statistical power to compare complication rates across settings, and no definitive conclusions regarding risk equality can be drawn.

## DISCUSSION

### Main results

Our study yields a striking epidemiological insight: ectopic pregnancy (EP) constitutes nearly one-quarter of all severe maternal morbidity (SMM) cases in Shenzhen, a megacity with a maternal mortality ratio (MMR) on par with the most developed nations. This finding reveals a crucial shift in the landscape of maternal health threats within advanced healthcare systems. As successes in preventing deaths from postpartum hemorrhage and hypertensive disorders accumulate, the relative burden of life-threatening early-pregnancy complications rises precipitously. Crucially, our analysis reveals that the pre-hospital and initial clinical encounter represent the most critical period of vulnerability, with three-quarters of EP-specific SMM cases originating from scenarios initiated at home or in primary/emergency care settings. This distribution highlights that the greatest systemic risks are no longer within the controlled environment of the obstetric ward but are concentrated at the fragmented interfaces between the community, the front line of healthcare, and the specialised referral center.

### Interpretation with literatures

This epidemiological transition we observed is consistent with, yet critically extends, the existing global literature. Studies from high-income countries have previously identified hemorrhage, particularly from ruptured ectopic pregnancy, as a leading cause of SMM and maternal intensive care unit admission [8,25,26]. Study conducted by Lisonkova, showed that while hospitalisation for ectopic pregnancy declined in Washington state between 1987 and 2014, severe maternal morbidity/mortality among hospitalised women increased [26].

However, the novel contribution of our work lies in its granular dissection of the resuscitation initiation scenario. Previous research, such as the seminal work by Leontine *et al*. on MMEIG, has excelled at quantifying and predicting the global incidence of SMM [27] but often lacks the contextual data to identify the precise clinical interfaces where acute deterioration occurs.

The study not only determines the incidence but also reveals a crucial pattern: the majority of acute crises appear before the patient is under definitive in-hospital monitoring. Our data show that 46.12% of EP-SMM cases had their emergency onset at home (potentially corresponding to Delay 1: delay in decision to seek care), and 30.20% occurred during the primary/emergency care phase (potentially corresponding to Delay 2: delay in reaching an appropriate facility or receiving timely initial management). In contrast, only 23.67% of cases originated during the in-hospital phase (Delay 3: delay in receiving adequate care after admission). We acknowledge that our study did not directly measure the components of each delay [28]; rather, we use this framework as a hypothesis-generating tool to contextualise our descriptive findings. The observed distribution suggests that Delays 1 and 2 are the dominant contributors to severe outcomes in our setting, whereas Delay 3, though less frequent, persists for complex cases. We specifically highlight that these proposed delay points, while our data supports them, require dedicated health services research with direct measurement of care-seeking behaviours and referral processes to be rigorously validated.

### Implications for public health policy

Our findings, although descriptive in nature, may offer valuable insights for strengthening maternal safety networks. The observed concentration of risk in the pre-hospital and primary care phases suggests that redirecting preventive efforts to these areas could be a promising strategy. We present the following policy implications as hypothesis-generating suggestions, based on our data, and intended for consideration by policymakers, rather than as definitive causal recommendations.

First, at the clinical level, the high proportion of home-onset shock calls for targeted public health campaigns to improve ‘early pregnancy literacy’, teaching women to recognise warning signs like acute abdominal pain and vaginal bleeding and to seek immediate medical attention. For primary care providers, our data support the mandatory implementation of a ‘Pregnancy Test First’ protocol for any woman of reproductive age with relevant symptoms, coupled with clear referral pathways for a positive result, as recommended by SOGC guidelines on managing EP [29]. Second, at the systems level, the high burden of cases originating in primary care/emergency departments argues for the creation of formalised ‘early pregnancy emergency networks’. Such networks could leverage telemedicine to provide immediate specialist consultation and ultrasound interpretation to frontline providers, effectively bringing expert care to the point of first contact. This model has shown promise in improving outcomes in other time-sensitive conditions like stroke and could be adapted for maternal health [30]. Finally, at the policy level, our findings suggest that MMR alone is an insufficient metric for systems like Shenzhen's. Monitoring the incidence of EP-specific SMM and, more proactively, tracking process measures like ‘time from first medical contact to diagnosis’ could serve as powerful sentinel indicators for auditing system performance, guiding resource allocation towards strengthening the weakest links in the chain of survival.

Crucially, the practical translational value of this research has already begun to materialise, moving beyond speculative recommendations into active health policy execution. Over an 8-month period following the identification of these structural vulnerabilities, our research team collaborated closely with the Municipal Health Commission to formally issue a city-wide policy directive titled<Guiding Opinions on the Management and Prevention of Ectopic Pregnancy>. This administrative framework directly addresses the frontline gaps highlighted in our cohort, specifically focusing on enhancing emergency department diagnostic accuracy, requiring standardised screening processes, and increasing community health efforts. To rigorously validate the hypotheses generated by this descriptive study, a comprehensive city-wide follow-up audit is scheduled for the end of this year. This upcoming evaluation will analyse municipal-level registry data to empirically assess whether these targeted system-wide interventions have achieved a significant reduction in catastrophic ectopic pregnancy rescue cases, thereby providing definitive evidence on our capacity to mitigate preventable adverse outcomes in this high-risk obstetric population.

### Limitations

Several limitations of this study must be acknowledged. First, its retrospective, registry-based design inherently relies on the completeness of routine electronic medical records, introducing potential information bias. Although our analysis covers a population-wide database across all 66 maternity hospitals in Shenzhen – which significantly strengthens generalisability – incomplete covariate ascertainment remains a challenge, and 12 cases (4.8%) had to be excluded due to missing data. Second, our study lacks a comprehensive, city-wide denominator for all diagnosed EP across the entire municipality, preventing the calculation of the exact proportion of EP that ultimately progress to EP-SMM. The registry is structurally designed to monitor births and severe near-miss events; consequently, uncomplicated EP managed successfully *via* medical therapy or expectant observation were not captured. Third, because this is an observational and descriptive audit without a control group of non-complicated ectopic pregnancies, we are precluded from making definitive causal inferences regarding the independent effects of socio-demographic characteristics on emergency locations. Fourth, our evaluation of clinical prognosis is heavily constrained by the extremely low event count for advanced severe complications (n = 7). Consequently, the study is statistically underpowered to detect subtle differences in rare secondary outcomes across presentation settings, and the lack of statistically significant differences must not be misinterpreted as proof of clinical equivalence. Fifth, critical patient-level clinical and behavioural risk factors, including history of fertility treatments, prior ectopic pregnancy, pelvic inflammatory disease, smoking habits, recent contraceptive use, and multidimensional socioeconomic indicators, were unavailable in our database. While our study maps vital systemic touchpoints, the observed severe outcomes must be interpreted as a reflection of both systemic challenges and these unmeasured individual complexities. Finally, the study setting in a high-resource Chinese megacity also limits the direct applicability of our findings to low-resource or rural settings where different barriers may predominate. Future prospective studies with comprehensive data capture and control groups are highly warranted.

## CONCLUSIONS

In conclusion, this study reveals that in a low-MMR setting, ectopic pregnancy accounts for a substantial proportion of severe maternal morbidity, with a striking concentration of emergency presentations occurring at home and in primary/emergency care settings. While our purely observational design precludes definitive causal attributions regarding specific systemic failures, the distinct spatial and clinical patterns identified across the 245 cases offer critical baseline insights. The striking concentration of hemorrhagic shock originating at home and in frontline healthcare settings suggests the need to refocus maternal safety strategies. Future public health initiatives should prioritise strengthening early recognition, community literacy, and rapid-response chains at the primary care level, complementing existing in-hospital protocols for late-pregnancy complications. These observational findings should serve as hypothesis-generating evidence to guide future prospective health services research and targeted operational interventions.

## Additional material


Online Supplementary Document

## Data Availability

**Data availability:** The data that support the findings of this study are available from Shenzhen Maternal and Child Health Care Management Information System but restrictions apply to the availability of these data, which were used under license for the current study, and so are not publicly available. Data are however available from the authors upon reasonable request and with permission of Shenzhen Maternal and Child Health Care Management Information System.
