# Supplementary material for: Ectopic pregnancy-related severe maternal morbidity in Shenzhen: a sentinel indicator for gaps in early pregnancy care systems
Source: J Glob Health. 2026 Oct 9;16:04331. doi: 10.7189/jogh.16.04331 (PMC13651763; doi:10.7189/jogh.16.04331)
Supplement: Supplementary file 1 [file jogh-16-04331-s001.pdf]

Supplement to: Dai Y, Wu Q, Zhu L, Yang W, Jin P, Jin S, Tian F, Li X. Ectopic pregnancy-related severe maternal morbidity in Shenzhen: a sentinel indicator for gaps in early pregnancy care systems. J Glob Health. 2026;16:04331.

Supplementary Data Sheet 1 Emergency Settings and Ectopic Pregnancy Types

Supplementary Data Sheet 2 Variation in ectopic pregnancy type distribution across clinical settings

Supplementary Data Sheet 3 Gestational age frequency of SMM-EP cases

Supplementary Data Sheet 4 Distribution of Gestational Age Among Different Types of Ectopic Pregnancies

Supplementary Data Sheet 5 Gestational age frequency of SMM-EP cases in different clinical settings

Supplementary Data Sheet 6 Geospatial distribution of SMM-EP incidence in Shenzhen

Supplementary Sheet 7 SMM indicators and corresponding ICD codes

Supplementary Data Sheet 1 Emergency Settings and Ectopic Pregnancy Types

	Isthmic p. (N=99)	Ampulla p. (N=76)	Interstitial p. (N=34)	Fimbrial p. (N=10)	Others（N=26）	F/X^2	P
Age	31.75±5.54	32.41±6.37	30.82±6.89	32.20±7.24	31.62±5.41	0.429[a]	0.788
Gestational age (weeks)	6.88±1.90	6.45±1.90	6.26±1.97	6.40±1.71	6.81±2.00	0.191 [a]	0.943
Gestational age (weeks)						3.941[b]	0.868
≤6	47（47.47）	42（55.26）	20（58.82）	6（60.00）	14（53.85）		
7-9	42（42.42）	31（40.79）	12（35.29）	4（60.00）	11（42.31）		
≥10	10（10.10）	3（3.95）	2（5.88）	0（0.00）	1（3.85）		
Gravidity						4.833[b]	0.784
1	17（17.17）	19（25.00）	6（17.65）	2（20.00）	7（26.92）		
2~4	66（66.67）	42（55.26）	20（58.82）	6（60.00）	13（50.00）		
≥5	16（16.16）	15（19.74）	8（23.53）	2（20.00）	6（23.08）		
Parity						3.928[b]	0.858
0	31（31.31）	27（35.53）	11（32.35）	3（30.00）	8（30.77）		
1~3	66（66.67）	46（60.53）	22（64.71）	6（60.00）	18（69.23）		
≥4	2（2.02）	3（3.95）	1（2.94）	1（10.00）	0（0.00）		
Educational Level						13.097 [b]	0.654
Junior High School/ Below	28（28.28）	21（27.63）	13（38.24）	6（60.00）	10（38.46）		
High School	23（23.23）	15（19.74）	4（11.76）	2（20.00）	4（15.38）		

College	25（25.25）	22（28.95）	12（35.29）	0（0.00）	8（30.77）		
Bachelor	22（22.22）	16（21.05）	5（14.71）	2（20.00）	4（15.38）		
Master and Above	1（1.01）	2（2.63）	0（0.00）	0（0.00）	0（0.00）		
Occupation						17.882 [b]	0.093
Housewife/Unspecified	34（34.34）	34（44.74）	20（58.82）	3（30.00）	6（23.08）		
Worker/Farmer	28（28.28）	13（17.11）	4（11.76）	3（30.00）	6（23.08）		
Merchant	5（5.05）	2（2.63）	3（8.82）	1（10.00）	3（11.54）		
Staff/Cadre	32（32.32）	27（35.53）	7（20.59）	3（30.00）	11（42.31）		
prenatal healthcare						17.098 [b]	0.008
0	95（95.96）	75（98.68）	33（97.06）	10（100.00）	20（76.92）		
≥1	4（4.04）	1（1.32）	1（2.94）	0（0.00）	6（23.08）		
Different settings						3.323 [b]	0.928
Home	46（46.46）	38（50.00）	13（38.24）	6（60.00）	10（38.46）		
Outpatient/ Emergency	30（30.30）	21（27.63）	11（32.35）	3（30.00）	9（34.62）		
Inpatient	23（23.23）	17（22.37）	10（29.41）	1（10.00）	7（26.92）		

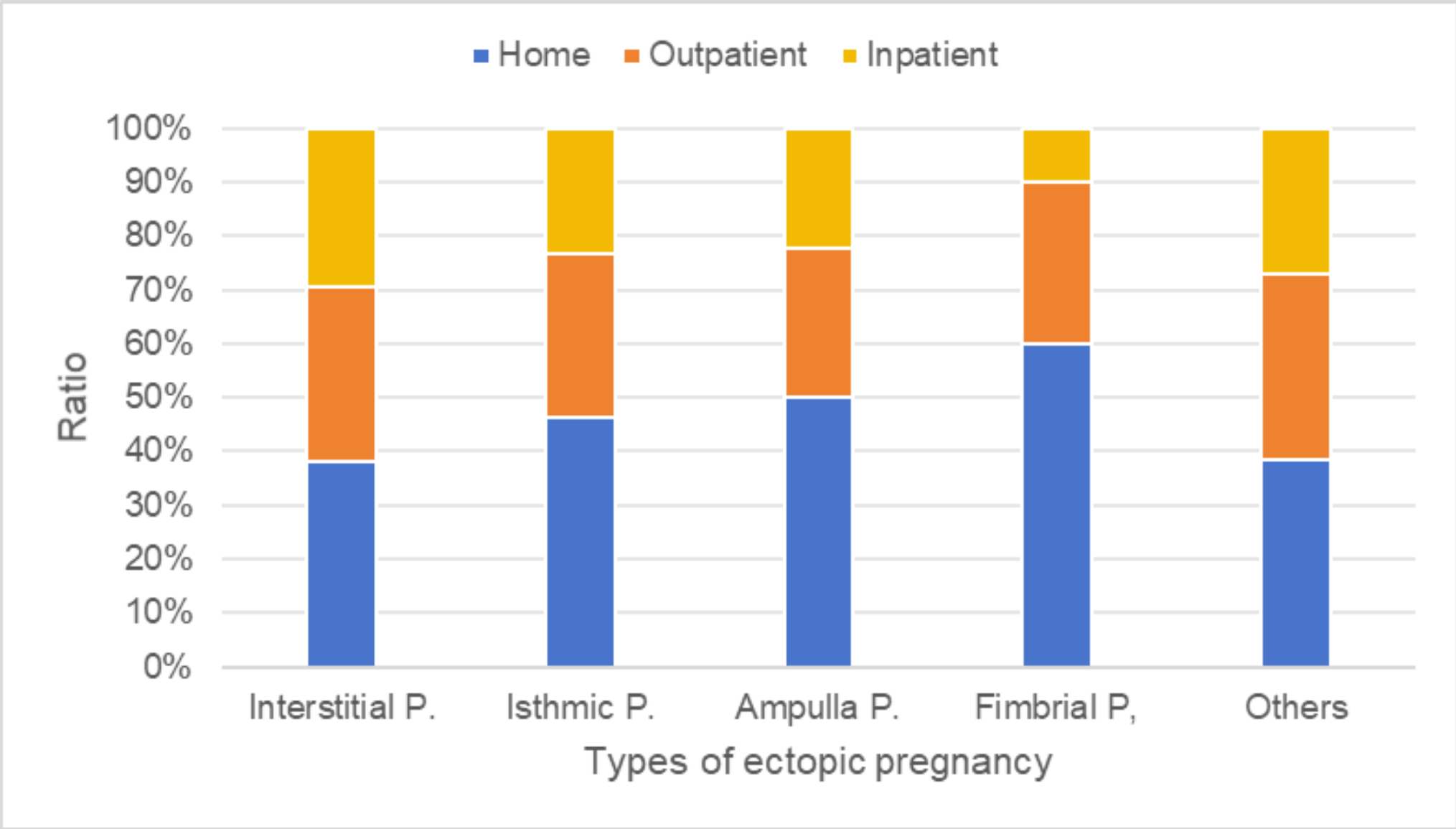

Supplementary Data Sheet 2 Variation in ectopic pregnancy type distribution across clinical settings

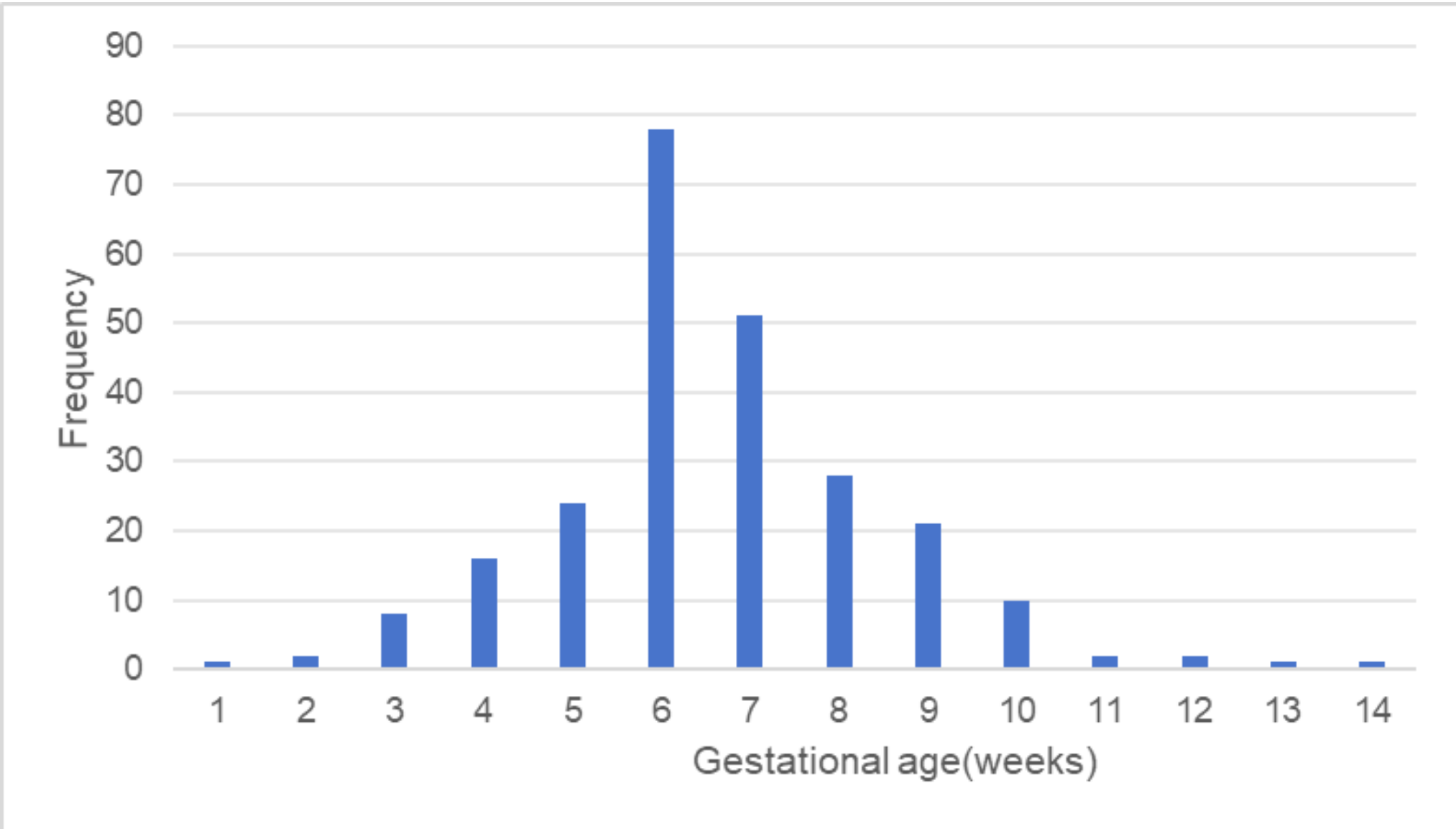

Supplementary Data Sheet 3 Gestational age frequency of SMM-EP cases

Supplementary Data Sheet 4 Distribution of Gestational Age Among Different Types of Ectopic Pregnancies

**Gestational age(weeks)**	**Interstitial P.**	**Isthmic P.**	**Ampulla P.**	**Fimbrial P.**	**others**
1	1	0	0	0	0
2	0	1	1	0	0
3	1	3	3	1	0
4	4	4	7	0	1
5	3	8	10	1	2
6	11	31	21	1	11
7	7	21	14	1	8
8	3	14	8	2	1
9	2	7	9	1	2
10	1	7	2	0	0
11	1	1	0	0	0
12	0	1	1	0	0
13	0	1	0	0	0
14	0	0	0	0	0
15	0	0	0	0	1
**Total**	**34**	**99**	**76**	**10**	**26**

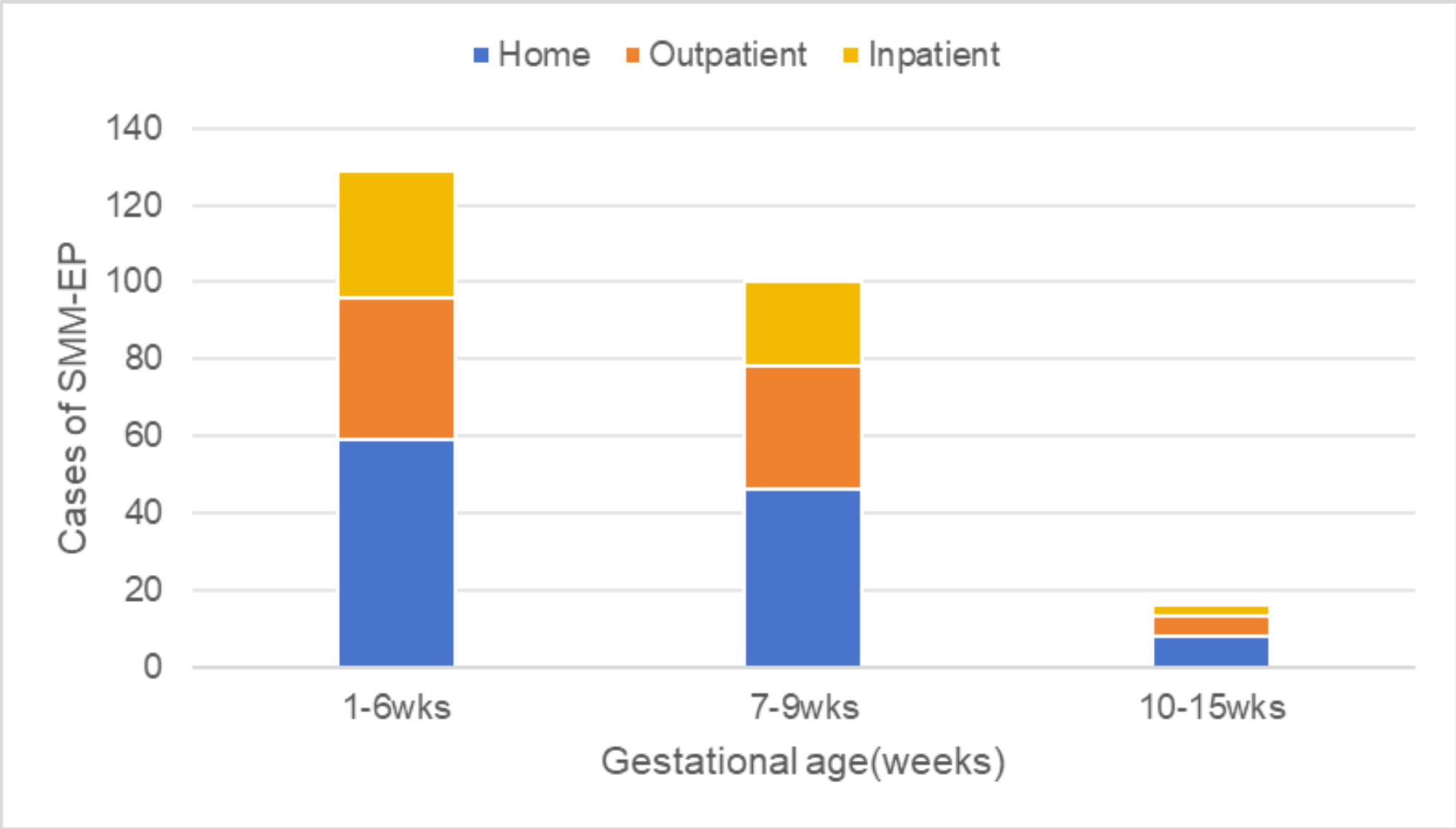

Supplementary Data Sheet 5 Gestational age frequency of SMM-EP cases in different clinical settings

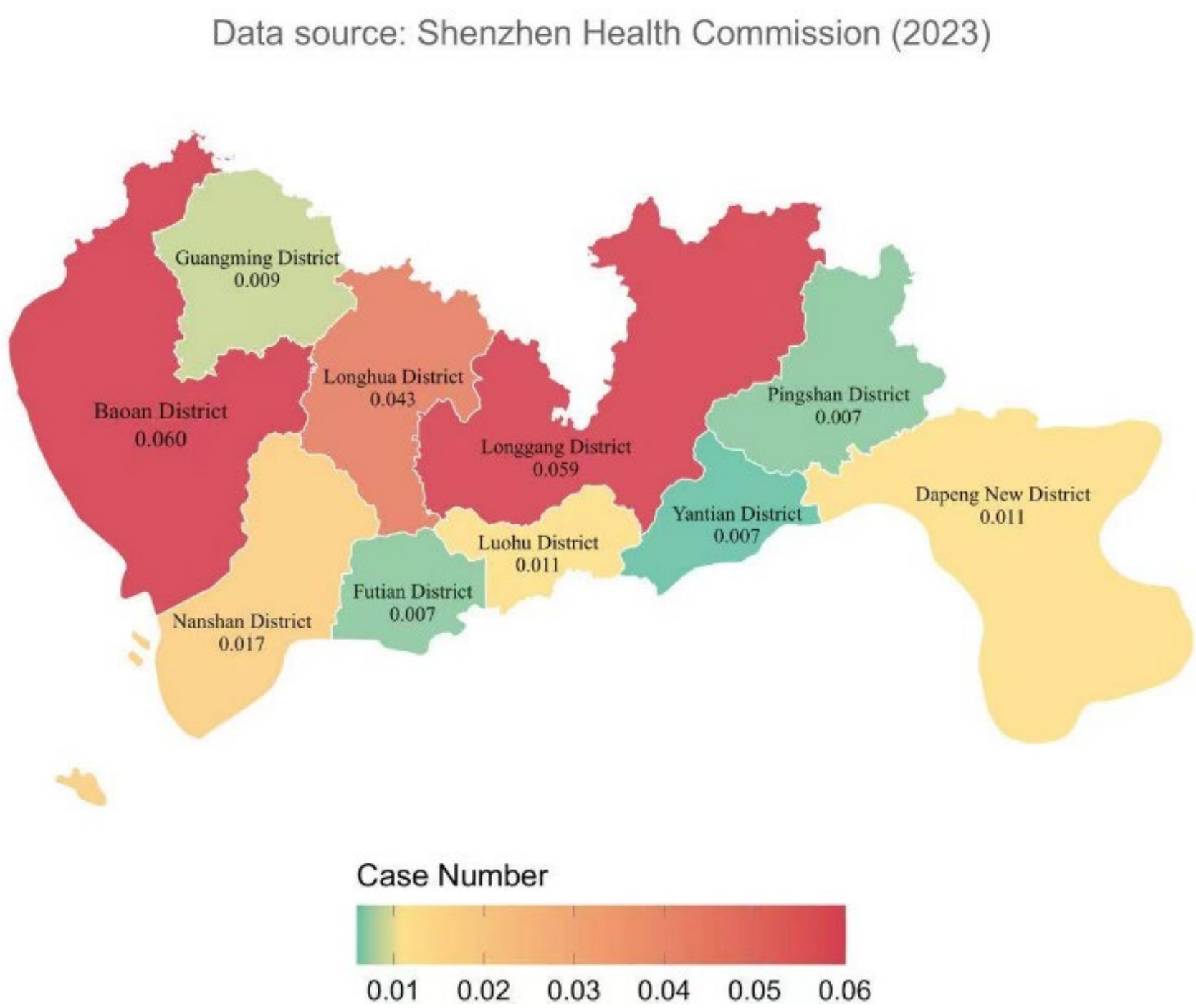

Supplementary Data Sheet 6 Geospatial distribution of SMM-EP incidence in Shenzhen

Supplementary Sheet 7 SMM indicators and corresponding ICD codes

**Severe Maternal Morbidity Indicator**	**ICD-10**
Acutecyanosis	R23.0，I73.800x008
Gasping	R06.200
**Shock**	O75.1, R57.x, T78.2XXA, T81.10XA, T81.11XA, T81.19XA, T88.6XXA
**Oliguria non responsive to fluids ordiuretics**	R34.x00，R34.01
Clotting failure	D68.9，D68.902，D68.900x007
Loss of consciousness lasting 12 hours	R40.200，R40.200x002，R40.201
Ectopic pregnancy **Indicator**	ICD-10: O00, O00.0, O00.1, O00.2, O00.8, O00.9
